# Pain as an Exploratory Marker of Rehabilitation Engagement After ACL Reconstruction: Combined Ligament Injuries and Digital Disengagement in a Sensor-Based Monitoring Cohort

**DOI:** 10.3390/jcm15124709

**Published:** 2026-06-17

**Authors:** Andreas Kopf, Wolfgang Hitzl, Christoph Bauer, Maximilian Willauschus, Johannes Rüther, Niklas Engel, Sophie Pennekamp, Lotta Hielscher, Vincent Franke, Hermann-Josef Bail, Markus Gesslein

**Affiliations:** 1 Department of Orthopaedics and Traumatology, Paracelsus Medical University, Breslauer Straße 201, 90471 Nuremberg, Germanymarkus.gesslein@klinikum-nuernberg.de (M.G.); 2 FIFA Medical Centre of Excellence, University Medical Centre Regensburg, 93053 Regensburg, Germany; 3 Research Office (Biostatistics), Paracelsus Medical University, Müllner Hauptstrasse 48, 5020 Salzburg, Austria; 4 Institute of Physiotherapy, School of Health Sciences, ZHAW Zurich University of Applied Sciences, 8400 Winterthur, Switzerland; 5 Lake Lucerne Institute, 6354 Vitznau, Switzerland; 6 Department of Internal Medicine 6, Paracelsus Medical University, Prof.-Ernst-Nathan-Straße 1, 90419 Nuremberg, Germany

**Keywords:** ACL reconstruction, knee surgery, collateral ligament, digital medical device, digital rehabilitation

## Abstract

**Background/Objectives:** To analyse postoperative pain trajectories after anterior cruciate ligament (ACL) reconstruction using data from a digital rehabilitation system, and to determine (i) whether combined ligament injuries are perceived as more painful than isolated ACL tears, (ii) which patient characteristics are associated with clinically relevant pain (visual analogue scale [VAS] > 5), and (iii) whether higher early pain is associated with later discontinuation of digital monitoring. **Methods:** This retrospective cohort study used routine data recorded by a validated sensor-based home rehabilitation system in patients after ACL reconstruction. This approach has previously been used to analyse functional recovery trajectories. All patients with ACL-related indications who performed at least one postoperative test were included and classified into four groups: isolated ACL rupture, ACL + meniscus, ACL + collateral ligament, and ACL + collateral ligament + meniscus. Pain during exercises and tests was recorded on a 0–10 VAS. High pain was defined as VAS > 5. Group comparison between indication types, anthropometric and activity-related variables and the proportion of high-pain events were performed using chi-square tests. Early pain (first postoperative month) was analysed in relation to the presence of later tests (≥3 months) to explore associations with discontinuation of digital monitoring. **Results:** Combined ligament injuries showed a significantly higher proportion of high-pain events during rehabilitation compared with isolated ACL ruptures (5.8% vs. 2.4%, overall *p* < 0.001). In particular, combined ligament injuries with ACL + collateral ligament rupture were associated with a greater share of VAS > 5 ratings in the early rehabilitation phases. No relevant association was observed between sex or BMI category and the occurrence of high pain, while age group showed an overall association without a consistent directional pattern. Sport activity level showed a strong relationship with high pain (*p* < 0.001). Early pain demonstrated a small but statistically significant negative correlation with later test participation (r = −0.15, approximately 2% of variance, *p* = 0.0076); however, this association was attenuated and no longer statistically significant when analysed using mixed-effects models accounting for within-patient clustering, indicating that patients with higher early pain tended to discontinue digital monitoring. **Conclusions:** Digital routine data after ACL reconstruction suggest that (i) combined ACL–collateral ligament injuries are perceived as more painful than isolated ACL tears, (ii) high postoperative pain is more closely related to activity level and injury pattern than to sex or BMI, while age group shows an overall but non-directional association, and (iii) higher early pain shows a weak bivariate correlation with digital disengagement that was not confirmed in mixed-effects models. Pain is therefore an exploratory marker warranting further investigation, rather than a confirmed independent predictor of adherence in app-based rehabilitation.

## 1. Introduction

Anterior cruciate ligament (ACL) injuries are among the most common sports-related knee injuries and frequently require surgical reconstruction [1,2]. While functional outcomes and return-to-sport rates after ACL reconstruction have been extensively investigated, the postoperative pain experience over the course of rehabilitation is significantly less described [3]. Most studies report pain at a few fixed follow-up time points or focus primarily on final functional outcomes, rather than on the longitudinal burden of pain during everyday rehabilitation exercises.

Digital, sensor-based rehabilitation systems have recently enabled continuous documentation of functional performance and patient-reported outcomes such as pain [4,5], offering a unique opportunity to characterise real-world recovery patterns across large, heterogeneous cohorts. In a previous study, we demonstrated that functional recovery after ACL reconstruction follows a non-linear pattern and that higher pain is associated with worse functional performance [5]. However, that analysis treated pain primarily as a covariate rather than as an outcome of interest. Several clinically relevant questions therefore remain unanswered: whether postoperative pain differs according to injury pattern, which patient-level factors are associated with clinically relevant pain, and whether higher early pain is associated with later disengagement from app-based monitoring. Rehabilitation after ACL reconstruction relies heavily on sustained engagement, and pain—as a dominant early postoperative experience—may influence not only physical performance but also behavioural responses such as motivation and willingness to repeatedly engage in rehabilitation tasks, making it a candidate early marker of patients at risk of reduced digital participation.

Using routine data from the Orthelligent sensor system by OPED in an ACL cohort, this analysis specifically focuses on pain as the central outcome.

Specifically, we investigated (i) whether pain intensity differs according to injury pattern, (ii) which demographic and activity-related factors are associated with clinically relevant pain, and (iii) whether early postoperative pain is associated with later digital disengagement.

We hypothesised that (i) ACL injuries with collateral ligament involvement are perceived as significantly more painful than isolated ACL ruptures, (ii) high pain is more strongly associated with activity level and injury pattern than with sex, age or BMI, and (iii) higher early pain is associated with a higher probability of later digital disengagement.

## 2. Materials and Methods

### 2.1. Study Design and Data Source

This study is a retrospective cohort analysis of routine data collected with a sensor-based home rehabilitation system (“Orthelligent home”, OPED, Valley, Germany) in patients after knee surgery. The same underlying dataset has previously been analysed to describe functional recovery patterns after ACL reconstruction [5]; the present work represents a targeted secondary analysis focusing on pain-related outcomes.

Patients were prescribed the digital system by their treating orthopaedic surgeon as an adjunct to standard physiotherapy or borrowed it without prescription for a fee. Using an inertial sensor and a smartphone application, patients performed predefined tests and exercises at home. During each event, the system recorded functional metrics (e.g., joint angles, jump height), contextual parameters (e.g., rehabilitation phase, days since surgery) and patient-reported outcomes including pain on a visual analogue scale (VAS, 0–10).

The database was generated and maintained by the device manufacturer (OPED GmbH, Valley, Germany), who provided the dataset for analysis under the terms of the research agreement (grant No. FMS_IF_006.25-I-2). The authors received a de-identified, read-only dataset; OPED had no role in data analysis, interpretation, or manuscript preparation. This is a multi-centre cohort: patients were recruited nationwide across Germany through prescriptions issued by individual treating orthopaedic surgeons in various clinical settings (both academic and private practice). No single centre contributed the majority of patients, and centre-level data were not captured in the dataset; this represents a limitation for centre-level confounding adjustment and is acknowledged in the Limitations section.

### 2.2. Study Population

The database contained all users of the system within the period between August 2022 and December 2024. For the present analysis, we included only (i) patients with a documented surgery date, (ii) who had undergone ACL-related surgery, and (iii) who performed at least one postoperative test or exercise with a recorded pain value. ACL-related indications were grouped into four clinically meaningful categories, which were: (i) isolated ACL rupture, (ii) ACL rupture with meniscus injury, (iii) ACL rupture with collateral ligament injury, and (iv) ACL rupture with combined meniscus and collateral ligament injury. Patients older than 65 years were excluded.

Inclusion criterion (iii)—requiring at least one recorded pain value—was applied because the present analysis is explicitly pain-centred; patients without any pain entry cannot contribute to the outcome of interest. The number of patients contributing only a single recorded event was 36 (10.7%), and a sensitivity analysis restricted to patients with ≥3 recorded events was performed to assess the influence of this group on the main results, with the results remaining directionally consistent. Regarding injury classification, ACL rupture is defined as a complete or partial ligamentous tear confirmed by MRI and/or intra-operatively; meniscal injury denotes any concomitant meniscal tear; and collateral ligament injury encompasses both medial (MCL) and lateral (LCL) ligament injuries as documented in the surgical report. The inability to differentiate MCL from LCL injuries is acknowledged as a limitation (see Limitations). The age groups 0–15 years (n = 4) and 61–65 years (n = 1) contribute negligibly to the overall sample. These patients were retained in the analysis to avoid post hoc exclusion bias, but their influence on grouped results is minimal and does not materially affect the conclusions.

### 2.3. Rehabilitation Programme and Phases

The rehabilitation algorithm and phase structure have been described previously [5]. In summary, all patients started in rehabilitation phase 0 immediately after surgery. Progression through phases 0–5 was determined by time since surgery and achievement of a minimum functional threshold (LSI ≥ 85%) to avoid overly rapid loading [5].

Because the focus of the present study was pain rather than functional performance, rehabilitation phases were additionally aligned with time since surgery to enable a uniform temporal analysis across different indication types. For this purpose, rehabilitation was divided into consecutive time windows covering the immediate postoperative period (days 0–20), early rehabilitation (days 21–45), intermediate phases (days 46–65 and 66–155), late rehabilitation (days 156–183), and periods beyond six months after surgery (>183 days) [5].

Pain intensity was assessed during each recorded test or exercise using a 0–10 visual analogue scale (VAS) within the app, where 0 represented no pain and 10 the worst imaginable pain. For analytical purposes, pain was operationalised in two complementary ways. First, early postoperative pain was treated as a continuous variable by calculating a patient-level mean VAS score based on all recorded events within the first postoperative month (0–1 month since surgery). Second, pain was analysed on an event level as a dichotomous outcome, with values above 5 classified as clinically relevant high pain and values of 5 or less considered low-to-moderate pain. The threshold of VAS > 5 was selected in accordance with commonly used clinical definitions and the original analysis plan [5].

Baseline patient characteristics available for analysis included sex; age group (categorised in 5-year intervals as recorded in the system); body mass index (BMI), which was continuous in the original dataset but categorised for selected analyses; pre-injury sport activity level as self-reported in the app; and indication type, comprising four ACL-related subgroups as described above. In addition, exercise-level data contained information on days since surgery, the rehabilitation phase at the time of each event, a unique patient identifier to link repeated measures, and indicators of rehabilitation engagement, including drop-out status, defined as the presence or absence of recorded tests beyond a specified postoperative time point.

### 2.4. Outcome

Based on the predefined research questions, three main outcomes were analysed. Firstly, pain burden was assessed by indication type, defined as the proportion of rehabilitation events with high pain (VAS > 5) within each indication group over the course of rehabilitation, with a particular focus on the early phases (phases 0–2). Secondly, determinants of clinically relevant pain were examined on an event level, with the occurrence of high pain (VAS > 5) as the outcome variable and sex, age group, BMI category, pre-injury sport activity level, and indication type as predictors. Thirdly, the association between early postoperative pain and digital disengagement was analysed at the patient level. Early pain was defined as the mean VAS score during the first postoperative month (0–1 month after surgery), while digital disengagement was operationalised as the absence of recorded tests from month 3 onwards. Patients without tests beyond month 3 were considered to have disengaged from the digital monitoring pathway, which does not necessarily imply discontinuation of all rehabilitation but rather cessation of app-based participation.

### 2.5. Statistical Analysis

IBM SPSS Statistics (Version 30.0, SPSS Inc., Chicago, IL, USA) and Microsoft Excel (Microsoft 365, Microsoft Corporation, Redmond, Washington, DC, USA) were used for the statistical analysis. All analyses were exploratory and based on predefined research questions.

1. Pain by indication type
  - Event-level data were used to calculate the proportion of high-pain events per indication group.
  - Group differences were tested using chi-square tests (Pearson or likelihood-ratio as appropriate). To account for the clustered structure of the data (multiple events nested within patients), mixed-effects logistic regression models with a random intercept for patient ID were additionally performed. High pain (VAS > 5) was modelled as the binary outcome, with indication type, pre-injury sport activity level, sex, age group, and BMI entered simultaneously as fixed effects. These variables were selected a priori based on clinical plausibility and availability in the dataset; they serve as covariates to adjust for confounding in the event-level model. No additional confounders were available in the routine dataset (see Limitations). The chi-square tests reported for each variable separately (Section 2, Determinants of high pain) are bivariate and serve a descriptive-screening purpose; the mixed-effects model provides the primary multivariable estimate. The analyses focused on the overall association; no adjustment for multiple testing was applied.
2. Determinants of high pain
  - Cross-tabulations were created for high pain (yes/no) versus sex, age group, BMI category, activity level and indication type.
  - For each cross-tabulation, chi-square tests were performed. Where expected cell counts were low, categories were collapsed.
3. Early pain and digital disengagement
  - For each patient, an early mean pain score within the first postoperative month was calculated.
  - A Spearman rank correlation between early mean pain and the binary variable “has tests ≥ month 3” (yes/no) was computed to quantify the relationship between early pain and later participation.
  - Additionally, early pain was compared between patients with and without later tests using standard inferential statistics (group comparison; exact test type depending on distribution). All tests were two-sided, and *p* < 0.05 was considered statistically significant.
  - In addition, a nonlinear model was fit to describe the relation between the number of completed rehabilitation tests and early pain (VAS, month 1). Model fit was expressed by the coefficient of determination.

The study was also registered with the research management of Paracelsus Medical University (FMS_IF_006.25-I-2; Regeneration) and it was determined by the Institutional Review Board (IRB-2025-08) that no ethics vote is necessary.

AI statement: Artificial intelligence (AI) tools were used to support the drafting and editing of this text. All final decisions on content, wording, and interpretation were made by the authors.

## 3. Results

The analysed cohort comprised the same ACL-reconstructed population as in the previous functional analysis, with 335 patients and 5675 test and exercise events recorded across the defined rehabilitation phases. All four ACL-related indication types were represented, with isolated ACL ruptures forming the largest group with 2320 test and exercise events and a smaller proportion of patients presenting with additional meniscus injuries (1762), collateral ligament injuries (538) or the combination of both (1055).

Anthropometric and demographic distributions (age, sex, BMI) as well as the temporal structure of the rehabilitation programme were therefore identical to those already reported for the functional outcomes (Table 1) [5].

Across the entire rehabilitation period, pain intensity declined non-linearly with time since surgery, with the highest pain burdens observed in the early phases and a progressive reduction towards later phases. When comparing indication groups, there was a significant overall association between injury pattern and the proportion of high-pain events (VAS > 5) during rehabilitation (chi-square test, *p* < 0.001).

**Figure 1:**
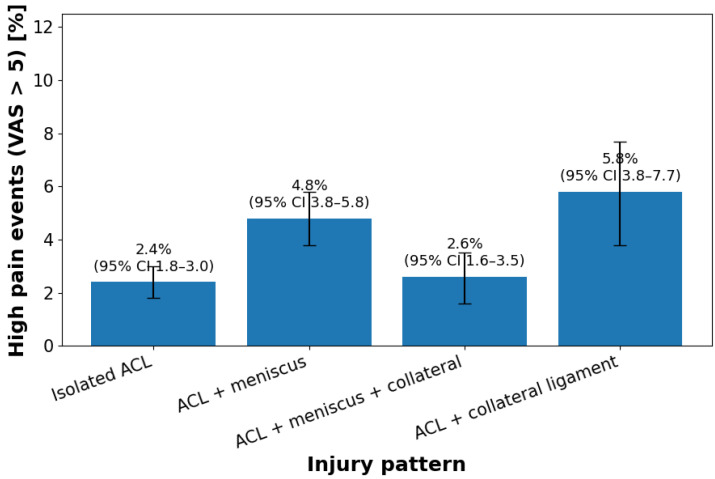
Proportion of rehabilitation events with high pain events (VAS > 5) stratified by ACL injury pattern across the entire rehabilitation period.

In line with the a priori hypothesis, indications involving collateral ligament injury (ACL + collateral) but also combined ACL + meniscus injuries showed a higher share of high-pain events compared with isolated ACL ruptures, particularly in the first postoperative months (Figure 1). While pain decreased over time in all groups, the relative excess of high pain in the collateral-involving group was most pronounced in early rehabilitation phases (approximately phases 0–2) and attenuated thereafter.

**Figure 2:**
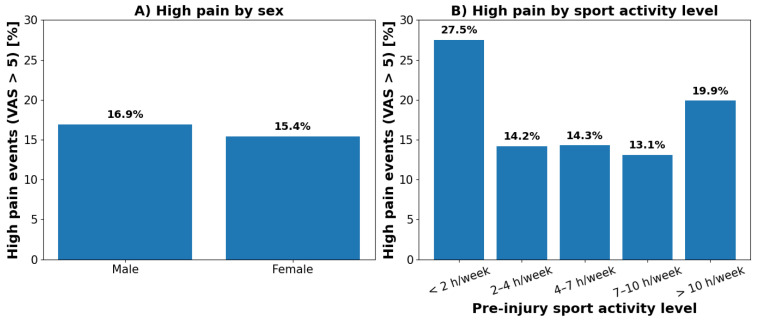
Determinants of high pain risk (VAS > 5) in males and females and by pre-injury sport activity level.

Event-level cross-tabulations revealed no statistically significant association between sex and the occurrence of high pain (VAS > 5). A chi-square analysis comparing the frequency of high pain between male and female patients yielded a non-significant result (*p* = 0.13) (Figure 2). A significant association was observed between age group and the occurrence of clinically relevant pain (VAS > 5). However, post hoc pairwise comparisons between age categories revealed an inconsistent pattern, without a clear gradient across younger or older age groups.

In contrast, pre-injury sport activity level was strongly associated with the proportion of high-pain events. Clinically relevant pain (VAS > 5) stratified by pre-injury sport activity level showed a strong association between activity level and pain occurrence (*p* < 0.0001), with the highest pain burden in patients with very low and very high activity levels (Figure 2).

To explore whether early postoperative pain is associated with later participation in the digital rehabilitation programme, each patient’s mean pain within the first month after surgery was calculated and related to the presence of tests from month 3 onwards.

**Figure 3:**
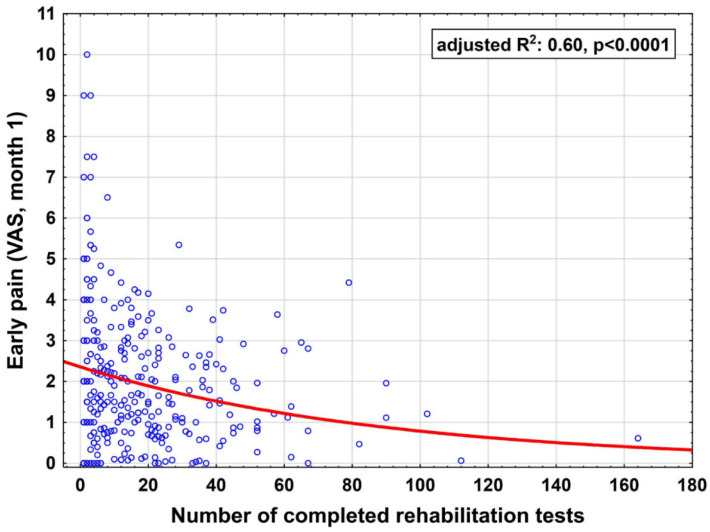
Scatterplot illustrating the association between mean pain intensity during the first postoperative month and the number of completed rehabilitation tests. A small but statistically significant negative correlation was observed (Spearman r = −0.15, *p* = 0.0076), explaining approximately 2% of the variance. The fitted trend line is shown for visual illustration only.

Early postoperative pain was inversely associated with later engagement in the digital rehabilitation programme. Spearman rank correlation analysis revealed a small but statistically significant negative correlation between mean pain during the first postoperative month and the number of completed rehabilitation tests (r = −0.15, *p* = 0.0076). This effect size corresponds to approximately 2% explained variance, indicating that early pain accounts for only a minor proportion of the variability in digital engagement behaviour. Patients with higher early pain scores tended to perform fewer digital assessments over time (Figure 3).

To account for the clustered data structure (multiple events per patient), a mixed-effects logistic regression model was additionally performed. When properly adjusting for within-patient correlation, the association between early pain and digital disengagement was attenuated and no longer reached conventional statistical significance. This suggests that the bivariate correlation, while descriptively present, may be partially attributable to pseudo-replication, and the independent predictive value of early pain for digital disengagement should be interpreted with caution.

## 4. Discussion

This secondary analysis of a large app-based ACL rehabilitation cohort provides three main insights regarding postoperative pain:

1. Combined ACL–collateral ligament or ACL–meniscus injuries are associated with a higher burden of pain during rehabilitation than isolated ACL ruptures, particularly in the early phases.
2. High pain (VAS > 5) is expected to be more strongly linked to pre-injury sport activity level and injury pattern than to sex, age or BMI, which appear to play a subordinate role in this context.
3. Higher early pain within the first postoperative month is associated with an increased likelihood of later digital disengagement, though the effect size is small (r = −0.15, explaining approximately 2% of variance) and should be interpreted cautiously.

The finding that combined ligament injuries, especially those involving the collateral ligaments, are perceived as more painful is clinically plausible and in line with the current literature [6]. Interestingly, as visible in Figure 1, injury patterns combining ACL and collateral ligament injury without concomitant meniscal damage showed a higher proportion of high-pain events than those involving both collateral ligament and meniscal injury—an observation that may initially appear counterintuitive given the greater anatomical complexity of the triple-structure group. Several explanations may account for this finding. Patients with ACL + meniscus + collateral ligament injuries may receive more restrictive early postoperative protocols (e.g., limited weight-bearing after meniscal repair), resulting in fewer high-load exercise events in the dataset during the early phase when pain is typically highest. Alternatively, the triple-structure group may represent a more heterogeneous population whose rehabilitation timing and intensity are individually tailored to a greater degree, attenuating the group-level pain signal. Finally, selection and reporting bias cannot be excluded: patients with the most complex injuries may disengage from digital monitoring earliest, leaving a survivorship sample with lower pain scores in the later time windows contributing to the aggregate proportion. These explanations remain speculative given the available data and should be tested in prospective studies. The present analysis may reflect differences in functional loading and rehabilitation demands rather than pure injury severity. Collateral ligament involvement, particularly on the medial side, has been shown to be associated with heterogeneous and prolonged responses to rehabilitation, with pain and functional tolerance varying considerably among patients and across protocols [7]. Limited standardisation in MCL rehabilitation and variabilities in mechanical stress tolerance may contribute to enhanced pain perception in isolated collateral injury patterns. These interpretations are supported by the biomechanical literature describing movement-pattern-dependent ligament loading and injury mechanisms [8]. An important limitation must be emphasised at this point: the present dataset does not allow differentiation between medial collateral ligament (MCL) and lateral collateral ligament (LCL) injuries. These structures differ considerably in their biomechanical function, loading patterns during rehabilitation exercises, and typical recovery trajectories. Their conflation within a single “collateral ligament” category may obscure clinically relevant differences in pain burden, and this should be given particular weight when interpreting the observed pain patterns and when comparing these findings with the existing literature on MCL- or LCL-specific outcomes. Beyond biomechanical considerations, the observed pain patterns may also reflect differences in surgical timing and early postoperative inflammatory burden. Combined ACL injuries involving collateral ligament damage are frequently treated earlier after trauma than isolated ACL ruptures, potentially during a phase of heightened inflammatory activity. This may contribute to increased pain sensitivity in the early rehabilitation period. Importantly, these findings suggest that patients with collateral ligament involvement should not necessarily be managed according to standard postoperative ACL rehabilitation protocols. Instead, individualised postoperative and rehabilitation strategies accounting for increased pain vulnerability may be warranted. This represents a key clinical implication of the present findings. Collateral ligament injuries are often associated with medial or lateral instability, soft-tissue trauma and extensive capsular irritation [9]. During early rehabilitation exercises—such as weight-bearing activities, single-leg squatting, balance and hopping tasks, and pivoting movements—these structures are subjected to varus/valgus stresses that may aggravate discomfort even if the ACL graft itself is adequately protected [5]. It should be noted that the present analysis treated rehabilitation events globally, without stratification by exercise category. Pain after ACL reconstruction is known to be task-dependent, typically highest during demanding weight-bearing tasks and lower during non-weight-bearing or range-of-motion exercises. Future analyses stratifying VAS scores by exercise type would allow identification of which specific movement categories drive the elevated pain burden in collateral ligament injury groups, substantially strengthening the clinical applicability of the findings.

From a rehabilitation standpoint, this underscores the need for injury-pattern-specific pain expectations: patients with complex ACL injuries should be counselled that a higher pain burden in the early weeks is common and does not necessarily indicate a complication but is crucial to address by adequate pain management since pain is associated with a higher rate of postoperative arthrofibrosis [10,11].

An important finding of the present analysis is the absence of a significant association between BMI and pain intensity, which is in line with the previously published literature [12]. While age and sex have been reported as relevant factors influencing pain perception in specific athletic populations, the present study did not demonstrate a consistent or directional effect of these variables on pain intensity, despite an overall association observed for age groups [13,14]. These discrepancies may be attributable to differences in study populations, sport-specific demands, or methodological aspects. In contrast, injury characteristics and the level of sporting activity emerged as the primary determinants of pain intensity in the present dataset.

The strong association between sport activity level and high pain might have several explanations. More active patients often push themselves harder in rehabilitation and may therefore provoke more discomfort during demanding tasks. They may be more aware of subtle symptoms and rate pain more strictly because they aim for high-level performance. Alternatively, their pain threshold or expectations regarding pain-free movement may differ from those of less active individuals.

Regardless of the exact mechanism, these observations highlight the importance of individualised pain counselling: in highly active patients striving for return to pivoting sports, early pain must be monitored carefully, but moderate levels of discomfort may be acceptable as long as function progresses and no red flags are present [15].

A clinically salient observation in the present dataset is the association between higher early pain (within the first postoperative month) and subsequent discontinuation of digital monitoring. Several important caveats must be emphasised upfront. First, digital disengagement as defined here—the absence of recorded tests from month three onwards—does not imply discontinuation of rehabilitation per se. Patients classified as digitally disengaged may have continued supervised physiotherapy, gym-based training, or sport-specific recovery programmes outside the app environment, a scenario that is particularly plausible in active athletic populations. The present findings therefore pertain exclusively to reduced participation within the digital ecosystem and should not be generalised to overall rehabilitation noncompliance. Second, the observed effect size was small (r = −0.15, explaining approximately 2% of variance), and critically, this bivariate association was attenuated and no longer statistically significant when within-patient clustering was accounted for in mixed-effects models. This methodological finding deserves strong emphasis: the independent predictive value of early pain for digital disengagement is not confirmed in the more appropriate analytical framework and should not be interpreted as evidence of a clinically meaningful predictor relationship. Other factors—including motivation, technological literacy, access to alternative care, and individual circumstances—likely play substantially larger roles. The finding should therefore be considered exploratory and hypothesis-generating rather than confirmatory evidence of a causal relationship. Nevertheless, the descriptive pattern aligns with the broader eHealth literature showing that attrition is common and should be treated as an outcome in its own right rather than merely a methodological inconvenience [16].

From a behavioural and clinical perspective, elevated early pain may act as a barrier to continued engagement through several potentially overlapping mechanisms: (i) avoidance of repeated exposure to painful tasks, (ii) reduced self-efficacy and perceived controllability of symptoms, and (iii) a shift in treatment preference toward more passive or clinician-delivered care when home-based exercise is experienced as overly aversive. Qualitative and mixed-methods work on home exercise programmes in musculoskeletal rehabilitation similarly identifies pain during exercise and the lack of perceived early improvement as prominent contributors to nonadherence [17]. More generally, rehabilitation adherence is strongly shaped by motivational constructs, supporting the plausibility that early pain—if interpreted as a threat or “something is wrong”—may undermine autonomous motivation and persistence [18].

In ACL reconstruction (ACLR) specifically, pain and related psychological responses are closely intertwined with functional recovery, and early symptom burden is likely to influence how patients appraise and execute rehabilitation tasks. Prior work has demonstrated that pain is consistently associated with function during different phases of ACLR rehabilitation, and fear of movement/reinjury becomes particularly relevant as patients progress toward higher-demand activities [19]. Contemporary syntheses further emphasise that psychological factors (e.g., kinesiophobia, fear of reinjury) are common after ACLR and can meaningfully impede recovery-relevant behaviours [20]. Importantly, digital ACLR rehabilitation studies highlight variable adherence and engagement, underscoring that platform-supported “self-rehabilitation” is feasible but vulnerable to disengagement—making early risk stratification clinically relevant [21].

Taken together, these findings support treating pain not only as a clinical symptom but also as an engagement signal in digitally supported ACLR rehabilitation. Consistent with the concept of a “science of attrition,” platforms should prospectively measure and report usage/retention metrics and integrate them with symptom trajectories to reduce informative missingness (i.e., the risk that those struggling most are also those most likely to disappear from follow-up) [16]. Practically, this could be operationalized through adaptive, safety-oriented design features, for example:

- Threshold-based clinician flags when pain remains above predefined levels across repeated sessions or worsens unexpectedly (to enable timely reassurance, analgesic optimisation, or load management).
- Dynamic exercise adaptation (progression/regression rules) in response to sustained high pain, analogous to graded activity principles.
- Targeted education and reassurance on expected postoperative pain fluctuations and the distinction between acceptable training discomfort versus warning signs, to reduce threat appraisals and avoidance.

This approach is also consistent with emerging evidence that digitally delivered programmes can capture granular patient-reported burden over time and that symptom fluctuations (including pain dynamics) can help identify individuals at risk of low adherence in exercise-based rehabilitation more broadly [22]. Finally, the present results extend earlier work linking pain to functional performance (e.g., LSI) by explicitly positioning pain as a primary outcome and demonstrating its relevance not only for physical trajectories but also for behavioural retention within a digital care pathway.

More broadly, our results are in line with reports that digital and telerehabilitation systems can capture nuanced patterns of patient-reported burden over time and identify critical “windows” for intervention [5]. It should be noted that the general concepts explored here—that combined ligament injuries produce greater postoperative burden, that pain may influence rehabilitation participation, and that digital rehabilitation systems experience attrition—are already relatively established in the literature. The genuine novelty of the present study lies not in the identification of entirely new mechanisms but rather in the longitudinal, app-based dataset with repeated real-world pain monitoring across a large cohort, enabling characterisation of pain trajectories with high temporal resolution. This contributes to a more comprehensive understanding of how subjective experience (pain) and behaviour (digital engagement) interact during sensor-supported ACL rehabilitation, a level of granularity not achievable with conventional clinic-based follow-up.

For clinicians, several practical messages emerge:

1. Set realistic expectations

Patients with ACL + collateral ligament injuries should be informed that they are likely to experience higher pain, especially in the early months, even with uncomplicated healing. This can prevent unnecessary anxiety and repeated consultations.

2. Monitor pain systematically

Pain should be tracked not only at clinic visits but also during home exercises, ideally using digital tools. Repeated high VAS scores should trigger review of exercise selection, dosage and analgesic strategies.

3. Identify at-risk patients early

Patients with high early pain and ambitious sport goals may be at particular risk of frustration and disengagement. Early targeted coaching, adjusted loading and closer follow-up could help maintain adherence.

4. Integrate pain into digital decision rules

Digital rehabilitation systems should incorporate pain thresholds and trajectories into their algorithms to personalise exercise progression and automatically flag potential disengagement risk.

### Limitations

This study has several important limitations that warrant careful consideration when interpreting the findings. First, the retrospective observational design using routine data precludes any causal inferences about the relationships identified. While we demonstrate associations between injury patterns, pain levels, and rehabilitation digital disengagement, the directionality and underlying mechanisms of these relationships remain uncertain. The correlation between early pain and disengagement, for instance, could reflect causation in either direction, or both variables might be influenced by unmeasured confounders such as psychological factors, social support, or access to alternative rehabilitation resources.

Second, substantial selection bias limits the generalisability of our findings. All patients in this cohort actively chose to use or were prescribed a specific digital rehabilitation system, representing a self-selected population that likely differs systematically from the broader ACL reconstruction population. Patients willing to engage with app-based rehabilitation may be younger, more technologically literate, more motivated, or have better access to healthcare resources than those who decline such systems.

Third, the definition of digital disengagement used in this study requires critical scrutiny. We operationalised disengagement as the absence of recorded tests from month three onwards, but this measure cannot distinguish between genuine cessation of rehabilitation and merely discontinuing use of the digital system while continuing conventional physiotherapy. Some patients classified as digitally disengaged may have achieved excellent functional outcomes through alternative rehabilitation pathways, while others may have discontinued all rehabilitation entirely.

Fourth, the absence of key clinical and contextual variables represents a structural limitation of the study design, not merely a consequence of using routine digital data. The dataset was not designed as a clinical research instrument and therefore does not capture perioperative pain management protocols, including the type and dose of analgesics prescribed, the use of peripheral nerve blocks, or pain management strategies recommended by treating surgeons. Postoperative pain responses after ACL reconstruction are known to differ substantially according to graft choice (e.g., bone–patellar tendon–bone vs. hamstring vs. quadriceps tendon), the performance of meniscal repair versus meniscectomy, postoperative weight-bearing restrictions, and the use of regional anaesthetic techniques—none of which are captured in the present dataset. The specific surgical techniques employed, concomitant procedures beyond those captured in our broad indication categories, and surgeon experience therefore remain unknown. Similarly, we lack information about concurrent physiotherapy intensity, frequency, and quality, which likely varies considerably across the nationwide, multi-centre cohort and could strongly moderate both pain experience and rehabilitation adherence. Psychological factors—including pain catastrophising, kinesiophobia, fear of reinjury, anxiety, and depression—are well-established determinants of both pain perception and rehabilitation adherence after ACL reconstruction and were not assessed in the present dataset. Without these parameters, it is not possible to determine with certainty whether the observed differences in pain trajectories reflect true injury-pattern effects or are partially attributable to differences in surgical protocols, analgesic management, or psychological burden across groups. These represent substantial potential confounders, and prospective studies with standardised clinical data collection are needed to adequately address them.

These omissions constitute potential confounders that could influence both pain intensity and digital engagement behaviour. Prospective studies with standardised clinical data collection are needed to adequately address these confounders.

Fifth, the grouping of injury patterns into four broad categories may obscure important within-group heterogeneity. For example, the severity and grade of collateral ligament injuries likely vary substantially within the “ACL + collateral ligament” group, yet all are treated identically in our analysis. Similarly, meniscal pathology ranges from small stable tears to large complex tears requiring extensive repair or resection, factors that may differentially affect pain trajectories but are not distinguished in our data structure.

Finally, factors that might confound the observed relationships were not available or controlled. These include pre-injury pain levels, chronicity of symptoms before surgery, previous knee injuries, psychological distress at baseline, pain coping strategies, patient expectations, and whether the injury was acute or chronic.

Despite these limitations, this study leverages the unique strengths of digital routine data to provide high temporal resolution insights into pain trajectories and adherence patterns in a real-world ACL rehabilitation cohort. The large sample size, longitudinal design, and integration of patient-reported and behavioural outcomes offer valuable hypothesis-generating findings that should be confirmed and extended in prospectively designed studies with more comprehensive clinical phenotyping and standardised follow-up protocols.

## 5. Conclusions

In a large cohort of ACL-reconstructed patients monitored with a digital home-based rehabilitation system, we found that:

- Combined ACL–collateral ligament injuries are associated with a higher burden of pain during early rehabilitation compared with isolated ACL ruptures.
- Clinically relevant pain (VAS > 5) is more strongly related to injury pattern and sport activity level than to sex, age or BMI.
- Higher early postoperative pain (first month) showed a weak bivariate correlation with digital disengagement (r = −0.15, *p* = 0.0076); however, this association was not confirmed in mixed-effects models adjusting for within-patient clustering. The finding is therefore exploratory and should not be interpreted as evidence that pain independently predicts disengagement. Prospective studies with comprehensive clinical phenotyping are needed to confirm or refute this hypothesis.

Pain may therefore be considered not only as a symptom to be treated, but also as a candidate exploratory marker of engagement behaviour in digitally supported ACL rehabilitation. The bivariate signal observed warrants further investigation in prospective, adequately powered studies with comprehensive clinical characterisation. Integrating systematic pain monitoring into rehabilitation pathways remains clinically meaningful regardless of its predictive value, as pain management directly affects patient experience and functional outcomes.

## Figures and Tables

**Table 1:** Anthropometric data and injury patterns of the population.

Group	ACL Injury (n = 134)	ACL + Meniscus Injury (n = 120)	ACL + Meniscus + Collateral Ligament Injury (n = 21)	ACL + Collateral Ligament Injury (n = 60)
**Height (cm)**	172.4 ± 8.7	177.9 ± 12.3	175.9 ± 8.8	172.0 ± 8.3
**Weight (kg)**	73.4 ± 14.7	75.4 ± 14.8	79.0 ± 14.6	71.5 ± 14.7
**BMI (kg/m^2^)**	24.7 ± 5.0	23.8 ± 4.7	25.5 ± 4.7	24.2 ± 5.0
**Age 0–15: n (%)**	3 (0.9%)	0 (0.0%)	1 (0.3%)	0 (0.0%)
**Age 16–20: n (%)**	14 (4.8%)	27 (8.1%)	9 (2.7%)	1 (0.3%)
**Age 21–25: n (%)**	30 (9.0%)	24 (7.2%)	8 (2.4%)	5 (1.5%)
**Age 26–30: n (%)**	25 (7.5%)	20 (6.0%)	10 (3.0%)	2 (0.6%)
**Age 31–35: n (%)**	14 (4.8%)	16 (4.8%)	4 (1.2%)	2 (0.6%)
**Age 36–40: n (%)**	16 (4.8%)	10 (3.0%)	8 (2.4%)	5 (1.5%)
**Age 41–45: n (%)**	30 (9.0%)	10 (3.0%)	11 (3.3%)	2 (0.6%)
**Age 46–50: n (%)**	4 (1.2%)	7 (2.1%)	5 (1.5%)	2 (0.6%)
**Age 51–55: n (%)**	3 (0.9%)	5 (1.5%)	2 (0.6%)	1 (0.3%)
**Age 56–60: n (%)**	1 (0.3%)	2 (0.6%)	2 (0.6%)	2 (0.6%)
**Age 61–65: n (%)**	0 (0.0%)	0 (0.0%)	1 (0.3%)	0 (0.0%)
**Sex distribution (F/M) (%)**	F = 46.4/M = 53.6	F = 40.5/M = 59.5	F = 45.9/M = 54.1	F = 45.5/M = 54.5

Percentages for age groups refer to the total study population (n = 335); within-group percentages can be derived by dividing the cell count by the respective group n shown in the column header.

## Data Availability

The data generated in this study are included in the results of the published article.
